# Unsupervised deep learning registration model for multimodal brain images

**DOI:** 10.1002/acm2.14177

**Published:** 2023-10-12

**Authors:** Samaneh Abbasi, Alireza Mehdizadeh, Hamid Reza Boveiri, Mohammad Amin Mosleh Shirazi, Reza Javidan, Raouf Khayami, Meysam Tavakoli

**Affiliations:** ^1^ Department of Medical Physics and Engineering School of Medicine Shiraz University of Medical Sciences Shiraz Iran; ^2^ Research Center for Neuromodulation and Pain Shiraz University of Medical Sciences Shiraz Iran; ^3^ Department of Computer Engineering and IT Shiraz University of Technology Shiraz Iran; ^4^ Ionizing and Non‐Ionizing Radiation Protection Research Center, School of Paramedical Sciences Shiraz University of Medical Sciences Shiraz Iran; ^5^ Department of Radiation Onc ology and Winship Cancer Institute Emory University Atlanta Georgia USA

**Keywords:** convolutional neural network, deep learning, medical image registration, unsupervised learning

## Abstract

Multimodal image registration is a key for many clinical image‐guided interventions. However, it is a challenging task because of complicated and unknown relationships between different modalities. Currently, deep supervised learning is the state‐of‐theart method at which the registration is conducted in end‐to‐end manner and one‐shot. Therefore, a huge ground‐truth data is required to improve the results of deep neural networks for registration. Moreover, supervised methods may yield models that bias towards annotated structures. Here, to deal with above challenges, an alternative approach is using unsupervised learning models. In this study, we have designed a novel deep unsupervised Convolutional Neural Network (CNN)‐based model based on computer tomography/magnetic resonance (CT/MR) co‐registration of brain images in an affine manner. For this purpose, we created a dataset consisting of 1100 pairs of CT/MR slices from the brain of 110 neuropsychic patients with/without tumor. At the next step, 12 landmarks were selected by a well‐experienced radiologist and annotated on each slice resulting in the computation of series of metrics evaluation, target registration error (TRE), Dice similarity, Hausdorff, and Jaccard coefficients. The proposed method could register the multimodal images with TRE 9.89, Dice similarity 0.79, Hausdorff 7.15, and Jaccard 0.75 that are appreciable for clinical applications. Moreover, the approach registered the images in an acceptable time 203 ms and can be appreciable for clinical usage due to the short registration time and high accuracy. Here, the results illustrated that our proposed method achieved competitive performance against other related approaches from both reasonable computation time and the metrics evaluation.

## 1 INTRODUCTION

**FIGURE 1:**
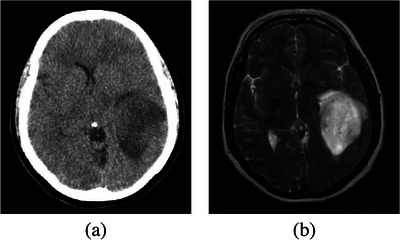
A sample of multimodal images for the registration from our local database. (a) Brain CT; (b) *T*_2_‐weighted MRI.

The brain tumor is one of the most dangerous and fast‐growing types of cancers.^1^ In this regard, image registration is a critical step for quantitative tumor evaluation. In clinical applications, image registration is necessary for various vital purposes, including diagnosis, prognosis, treatment, and follow‐up of cancer patients.^2^ For this purpose, multimodal magnetic resonance imaging (MRI) and computed tomography (CT) are the primary methods for screening and diagnosis. Multimodal registration aims to correlate relevant information from different clinical imaging scans.^3^ It is an important step to guide the multimodal information fusion and eventually simplify the clinical diagnosis and treatment. It can compensate for patient motion‐induced deformations caused by different positioning or breathing levels, as well as pathological variations among different imaging modalities. Figure 1 illustrates two typical multimodal brain images that require registration for clinical purposes.

However, multimodal image registration poses significant challenges in medical imaging, especially in the context of image‐guided intervention and data fusion.^4^ For instance, in radiotherapy, a fully marked and annotated CT image is inputted into the Treatment Planning System (TPS) for treatment planning.^5^ Here, although CT images alone may not provide complete differentiation of soft tissues such as tumors, they often need to be fused with MRI to aid physicians in the process of marking and annotating, enabling more accurate treatment with less invasive procedures.

Traditionally, image registration has been performed manually by physicians, which is a time‐consuming process and heavily reliant on the expertise of the user.^6^ Consequently, there is a need to explore automatic registration approaches. To address these challenges, various methods have been proposed, aiming to develop tools and algorithms for automated and accurate brain image registration and tumor segmentation.^7^ In this context, the annual Multimodal Brain Tumor Segmentation Challenge^8^ serves as a platform to encourage the development of efficient methods and provide insights into tackling this problem.

In radiotherapy treatment planning, affine registration is employed as a geometric transformation technique that preserves lines and parallelism, facilitating improved delineation of tumor boundaries. Given that CT imaging, used as an input for the TPS, is not ideal for visualizing soft tissue, it is commonly fused with MRI, which offers better soft tissue contrast. This fusion enables the acquisition of additional information from the images and enhances the accuracy and precision of tumor contour delineation.

In our current study, we plan to implement a deep unsupervised learning model for image registration. This approach will involve multimodal CT/MR affine co‐registration, utilizing a convolutional neural network (CNN) with mutual information (MI) loss functions. The aim is to achieve more precise image registration and alignment. Contributions and highlights of this work include:

1. A new differentiable layer is suggested to transform the affine transformation generated by the network into a dense displacement field. This affine‐to‐field layer allows us to apply various regularization techniques to the transformed output, helping to manage overfitting during training while also increasing the depth of the network.
2. The proposed method is assessed using a challenging annotated multimodal dataset consisting of 1100 pairs of CT/MR brain images. The dataset exhibits various heterogeneities, such as differences in scanners, imaging setups, and resolutions. For instance, the training and testing data include a combination of *T*_1_ and *T*_2_‐weighted MR. The goal here is to assess how well the proposed model can handle and address the complexity arising from such diverse characteristics in the dataset.
3. The outcomes of the study demonstrate that the introduced customized layer effectively addresses the issue of overfitting in deeper networks. These deeper networks have the capability to generate more intricate transformations compared to shallower networks. This success is evident in the evaluation metrics, including the dice coefficient and target registration error (TRE).

The rest of the paper is organized as follows. In the next section we review some related published studies. Section 3 explains the proposed method with details of all processing and network architecture. Section 4 introduces the material and databases used in this study and presents the results. The final sections present a discussion and conclusion.

## 2 STATE OF ARTS

Many approaches have been proposed about image registration. Some comprehensive reviews on existing methods are presented.^6^, ^9^, ^10^ In general, registration algorithms can be classified into three broad categories: registration via optimization,^11^, ^12^ registration via statistical learning,^13^ and registration via deep learning (DL).^14^ The first one, via optimization, is based on deformable registration,^4^, ^8^ including the different classes, intensity‐based^15^ and feature‐based,^16^ and can be also based on linear rigid/affine registration. In the statistical learning registration, the models have been used to improve the performance by setting up the correlation between the deformation field and images based on training images. Here, the well‐known registration algorithms are support vector machine,^17^ sparse representation,^13^ and dictionary‐based method.^18^

Although other approaches for automatic image registration have been broadly investigated before or during exist of the DL, using DL has changed the viewpoint of image registration research. DL approaches such as the CNN have been demonstrated to be applicable for registration.^19^, ^20^ In general, the DL‐based methods are categorized into two classes: supervised and unsupervised learning. For supervised learning, Sokooti et al.^21^ introduced RegNet to estimate the displacement vector field for a pair of CT images. Cao et al.^22^ applied an equalized active‐points sampling algorithm to create a similarity‐steered CNN model to estimate the deformations points. Yang et al.^23^ worked on the estimation of the momenta of the deformation in a large deformation diffeomorphic metric mapping setting. Rohe et al.^24^ established reference deformations trained by manually registering the regions of interest. In the same direction, Hu et al.^25^ and Xu and Niethammer^26^ presented studies where networks were trained with the objective of maximizing the alignment between tissue labels. All supervised learning‐based registration methods require significant effort in constructing reference deformations, as the availability of ideal ground‐truth deformations for training is limited. This reliance on ground‐truth data poses a major challenge, as it necessitates expert labeling, which can introduce biases into the models and may not fully capture the desired deformations accurately.^27^, ^28^ To address these limitations, a deep unsupervised model is implemented, eliminating the need for ground‐truth data. This approach leverages neural networks such as generative adversarial networks (GAN), CNN, and spatial transformation networks (STN) to perform image warping. Instead of relying on ground‐truth data, unsupervised methods utilize similarity metrics as guiding signals.^6^, ^29^, ^30^ Considering the labor‐intensive nature of image registration when relying on annotating ground‐truth data, unsupervised methods offer a more suitable alternative.^31^

For unsupervised learning, some studies presented an end‐to‐end network to predict deformable transformations.^30^, ^32^, ^33^, ^34^, ^35^ In the same realm, this utilization was explored in studies conducted by Wu et al.,^36^ Cheng et al.,^37^ and Simonovsky et al.^38^ Here, two significant factors driving the adoption of DL include its efficiency in automated feature extraction, which enhances performance, and the presence of a local optima problem inherent in multimodal dissimilarity metrics. de Vos et al.^30^ employed a transformer networks to implement to achieve unsupervised end‐to‐end training for deformable image registration. Balakrishnan et al.^33^ introduced the VoxelMorph registration model, which utilizes a Ushaped CNN architecture. The VoxelMorph demonstrated a significantly faster registration speed in compare to some other learning approaches. Kuang et al.^39^ highlighted that the Folding‐Aware Image Mover's model can enhance the smoothness of the displacement vector field by incorporating a folding penalty regularization term in the objective function. This approach yielded improved registration outcomes while requiring fewer parameters compared to VoxelMorph. Fechter et al.^40^ presented a registration network based on one‐shot learning, designed to effectively handle the variability among different training datasets. Kim et al.^41^ improved the registration performance of CycleMorph by incorporating cycle consistency as an implicit regularization, ensuring the preservation of topology in the deformation field. Meanwhile, de Vos et al.^42^ introduced a DL framework based on a coarse‐to‐fine approach, achieving high accuracy in chest‐CT registration. Furthermore, Fan et al.^43^ developed a registration network using a GAN model, where a discriminant network served as an adaptive similarity measure to train the network. Zhou et al.^44^ introduced a domain adaptation network called the anatomy‐preserving domain adaptation network. This network has the capability to learn the representation of anatomical structures even in the absence of groundtruth data.

In fact, unsupervised methods aim to maximize the image similarity between a pair of images without relying on ground‐truth deformations. These methods utilize estimated similarity metrics such as the sum of squared difference or cross‐correlation to train the registration network. However, these metrics heavily rely on assumptions about the relationship of image intensities and may not always provide optimal results.^14^ Additionally, many of these methods optimize an energy function for each image pair, leading to slow registration.

In this study, we focus on multimodal CT/MR affine co‐registration using a deep unsupervised model based on CNN with MI loss functions. Our approach involves comparing *T*_1_ and *T*_2_‐weighted MRI of the brain and performing inter‐subject deformable registration, which involves comparing all images with an atlas. Furthermore, we also address intrasubject registration, known as co‐registration. It is worth noting that the implementation of MI as a differentiable function contributes to reducing the complexity of the registration process.

## 3 PROPOSED METHOD

**FIGURE 2:**
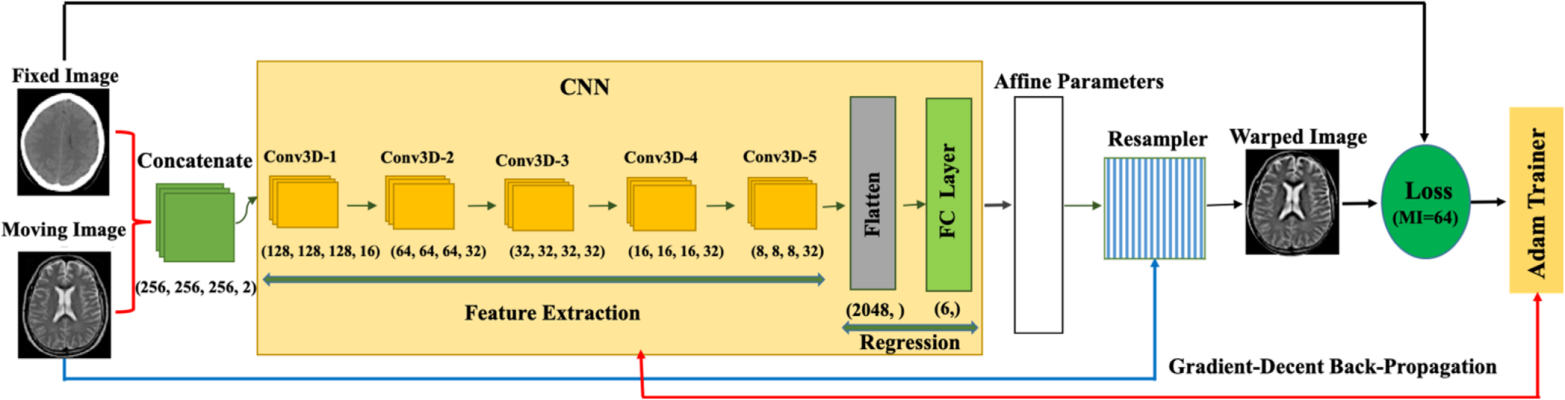
The schematic workflow diagram of the proposed model.

**FIGURE 3:**
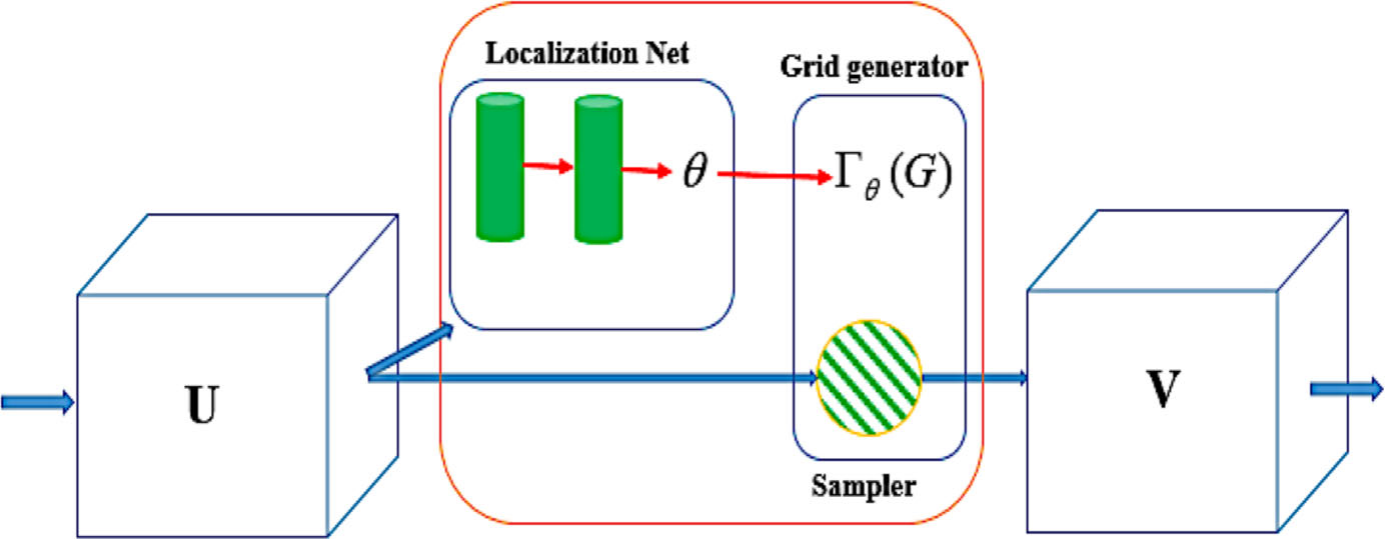
The architecture of the STN for the registration purpose.

The schematic diagram of our workflow and network is sketched in Figure 2. In the current study, the proposed model is designed based on an innovative technique within deep neural networks called regularized STN (Figure 3),^29^ that is, the transformation parameters are provided using a CNN network as the localization network, provides the transformation parameters (here, six parameters for the affine transformation), and a combination of grid‐generator and resampler applies the provided transformation to the moving image in order to reproduce the warped one. The major novelty brought by the STN was the introduction of a resampler layer that is differentiable. This crucial feature enables the training of the network through gradient‐descent back‐propagation, which marked significant progress in the field. Instead of relying on expensive and often limited ground‐truth data for the learning phase, the STN utilizes a differentiable implementation of a conventional similarity metric, such as local cross‐correlation for unimodal registration or normalized MI for multimodal registration. This differentiable metric serves as a substitute for ground‐truth, allowing the paradigm to be referred to as unsupervised learning when there is a lack of explicit ground‐truth information.

The main challenge in utilizing this architecture specially for the affine transformation lies in incorporating a regularizer module that penalizes unrealistic transformations generated by the network. This module plays a crucial role in achieving stable registration and preserving the structural integrity of flexible organs. However, the issue arises because the regularizer terms cannot be directly applied to the encrypted set of parameters representing the affine transformation. It has been observed that the regularizer can only be effectively applied to transformation fields.^45^

The primary innovation of the proposed approach lies in the base network, where the affine transformation obtained as output is directly applied to the moving image through the resampler layer, resulting in a warped image. However, this direct application of the affine transformation poses a challenge in incorporating a regularizer module into the model. The transformation is encoded within a matrix size *K* × *K*, where K represents the number of dimensions in the image. Affine registration, being a global transformation, requires deeper networks where the final layers can capture larger contextual information of the object of interest for alignment. Unfortunately, deeper networks, which are larger and more complex models, increase the risk of overfitting due to the limited degrees of freedom provided by the encrypted transformation. Consequently, the regularizer module plays a crucial role in enabling the development of deeper networks and mitigating overfitting during training. In this study, an Affine‐to‐Field layer is proposed to decrypt the transformation output from the network, which originally has a shape of (6,), into a displacement field with a shape of [256, 256, 256, 2] (2‐channel 3D input). This transformation allows for the application of different regularization terms, thereby facilitating the regularization process.

Let's assume the fixed and moving images as *F* and *M*, respectively, and the transformation function parametrized by *μ* that can be shown as *T_μ_*. Thus, the application of *T_μ_* on *M* leads to align it on *F*, that is, *T_μ_* : *M* → *F*.

(1) $$\mu b=\min{L_{g}}_{\theta }\left(T_{\mu }:M\to F\right),$$

where *g_θ_* is the CNN, that is, the model g parametrized by *θ*. In addition, a similarity metric *L* is considered as the loss function for conducting the training phase of the network. The stochastic gradient descent with back‐propagation algorithm is carried out on the network *g* so that the similarity metric *L* is minimized. The MI, as one of the most valid metrics for multimodal image registration, is used as a loss function of the proposed model. It is computed using the following formula:

(2) $$MI\left(F,M\right)=\sum_{f}\sum_{m}p\left(f.m\right)\log\left(\frac{p\left(f.m\right)}{p\left(f\right)p\left(m\right)}\right)$$

Here, *f* and *m* are the intensities of voxels in the *F* and *M*, respectively. Probabilities *p*(*f*) and *p*(*m*) are defined as the voxels proportion in image *F* and *M* with intensities *f* and *m*, respectively, computed via drawing a histogram of intensities of voxels for *F* and *M*.

The introduced unsupervised deep learning‐based models based on the STN encompass three major components, as follows: (1) A localization network takes the input feature‐maps (pair of inputted images) and estimates the transformation parameters in one‐shot. (2) A grid generator performs necessary preprocessing to apply the parameters on the inputted images and generates the final displacement field. (3) A resampler module creates the warped image by applying the generated displacement field on the moving image (see Figure 3). The key novelty of the STN lies in the differentiability of the resampler module, which allows us to train the network using the well‐known stochastic gradient‐descent with back‐propagation algorithm.

Since the inputted images (CT & MRI) are multimodal, their intensity distribution functions are completely independent. Additionally, the intensity range of the images in our dataset is broad due to variations in scanners, manufacturers, and radiology procedures. Consequently, the convergence of the network becomes challenging during the training process due to the wide range of inputted data. Therefore, as a preprocessing step, all CT and MR images in the dataset are normalized based on the mean and standard deviation of all pixel intensities before being inputted to the network.

### 3.1 Network architecture

As presented in Figure 2, the proposed model encompasses five consecutive modules including a concatenator, a 3D‐CNN as the localization network, a combination of grid‐generator and resampler, a customized loss function to conduct the training process (here, a 64‐bins MI), and finally an Adam optimizer to conduct the stochastic gradient‐descent with backpropagation algorithm. It is worth mentioning that the loss function and Adam optimizer are only applicable in the training phase since the reproduced warped image is the final artifact in the registration phase.

Here, a pair of fixed and moving images are inputted to the concatenator in order to be normalized and to construct a single 2‐channel input. The input size was 256 × 256 × 256 × 2, but the workflow is not limited by a certain size. In the localization network (CNN), five 3D successive regular convolutional layers with stride two are utilized with the numbers of filters as follows: [32, 32, 32, 32, 32]. The flatten layer vectorized 2048 parameters which was produced via the last convolutional layer. Finally, there is a fully‐connected layer with 2048 × 6 weights to generate the concluding parameters of the affine transformation. The concluded affine transformation is then applied to the moving image using the re‐sampler module to construct the warped image which is the final output for the registration phase. The architectural and learning hyper‐parameters of the proposed network are presented in Table 1.

**TABLE 1:** The architectural and learning hyper‐parameters of the proposed network.

Item	Proposed model	Baselines
No. of convolutional layers	5	5
Architecture	[32, 32, 32, 32, 32, 6]	[16, 32, 32, 32, 32, 6]
Total parameters	49,894	44,982
No. of MI bins	64	32 and 64
No. of epochs	500	500
Stride	2	2
Pooling layers	–	–
Layer initializer	He Normal	He Normal
Activation function	Leaky‐ReLU (0.2)	Leaky‐ReLU (0.2)
Dropout	–	–
Optimizer	Adam	Adam
Learning rate	10^−4^	10^−5^ and 10^−4^

Two important parts of the architecture are the resampler layer and the loss function. The gradient‐descent back‐propagation algorithm requires to compute back‐ward dataflow gradient for each layer to train the network in the routine manner.^46^ Moreover, here the system was implemented using Keras with TensorFlow‐GPU backend on an Nvidia GeForce GTX 1060 OC version with 6GB DDR‐V memory. The configurations and underlying platform in details are listed in Table 2.

**TABLE 2:** The configurations and underlying platform in detail.

Item	Value	Version
Platform	Microsoft Windows	10 × 64
CPU	Intel i7‐3770k	3.9 GHz
RAM	DDR III 12 GB	1600 MHz
GPU	Nvidia GTX 1060 OC 6GB	1811 MHz
Environment	PyCharm	2019.3.4
Library	Keras	2.2.4
Backend	Tensorflow‐gpu	1.12.0
	CUDA	9.0
	cuDNN	7.6.5

### 3.2 Implementation details

**FIGURE 4:**
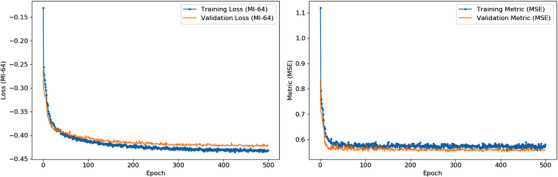
The proposed approach training with LR = 1E‐4.

For the evaluation purpose, three different baselines, Symmetric Normalization (SyN),^32^, ^47^ NiftyReg package,^48^, ^49^ and VoxelMorph,^35^ were investigated and their results were compared with the proposed method. Here for SyN, we applied its implementation in the available Advanced Normalization Tools (ANTs) software package.^50^ Throughout our study, we discovered that the default smoothness parameters of ANTs were sub‐optimal for our images. Furthermore, we implemented VoxelMorph using Keras with a TensorFlow backend.^51^ The Learning Rate (LR) in ANT (SyN) equals 10^−5^ and for other two methods and the proposed approach the LR increases to 10^−4^ as the learning process is more stable and the final network's performance is far better with such LR. As seen in Figure 4, increasing in LR results in better performance as the validation loss approaches the training loss. Moreover, number of parameters in all baselines are 44982.

### 3.3 Training process

Given that inputted images were in the shape of (256 × 256 × 256), the patch method was not used in this study and all images were completely provided to the network. Besides, data augmentation was not used as we were going to train the network with real images. Here we used 60 patients for training purpose, 20 patients for validation and 30 patients for the test data. The next section elaborates our strategy for data gathering and annotating.

## 4 EXPERIMENTAL RESULTS AND EVALUATIONS

### 4.1 Data collection

In this work, our dataset was randomly obtained from the neuropsychic patients from the hospital, with an Ethic code, using their native PACS called INFINIIT. We collected the images from 110 patients, and for each patient we had 10 slices for CT and 10 slices for the MRI total of 1100 pairs of slices, with and without tumor. According to the objective of the study, our goal is to perform image registration on images from two modalities: CT and MR. However, patients who underwent MR and CT scans on different dates were deemed unsuitable for image registration and were therefore excluded. This was necessary because patients who had undergone surgery or experienced significant changes in tumor morphology would introduce confounding factors. Hence, it was crucial to ensure that both the MR and CT scans were acquired at approximately the same date for each patient. Furthermore, we made an effort to select images randomly and appropriately, ensuring a balanced distribution of CT scans and MR images (with and without tumors) across the dataset in three classes, including training (*n* = 60), validation (*n* = 20), and test (*n* = 30) sets.

All CT and MR images (including both *T*_1_ and *T*_2_ weighted) were randomly taken from various scanners with different manufacture, setups, and procedures. Thus, there was heterogeneity in the dataset resulting in a multimodal and more challenging co‐registration study that the proposed model could address this challenge properly. Meanwhile, since the images used in this study have different resolutions, including (480 × 480 × 480), (384 × 384×384), (256×256×256), and (521×521×521), we preferred downscaling all of them to 256 × 256 × 256 as the present study is based on the affine image registration that the high resolution does not have a significant impact on the results,^52^ but it prolongs the training time fantastically.

**FIGURE 5:**
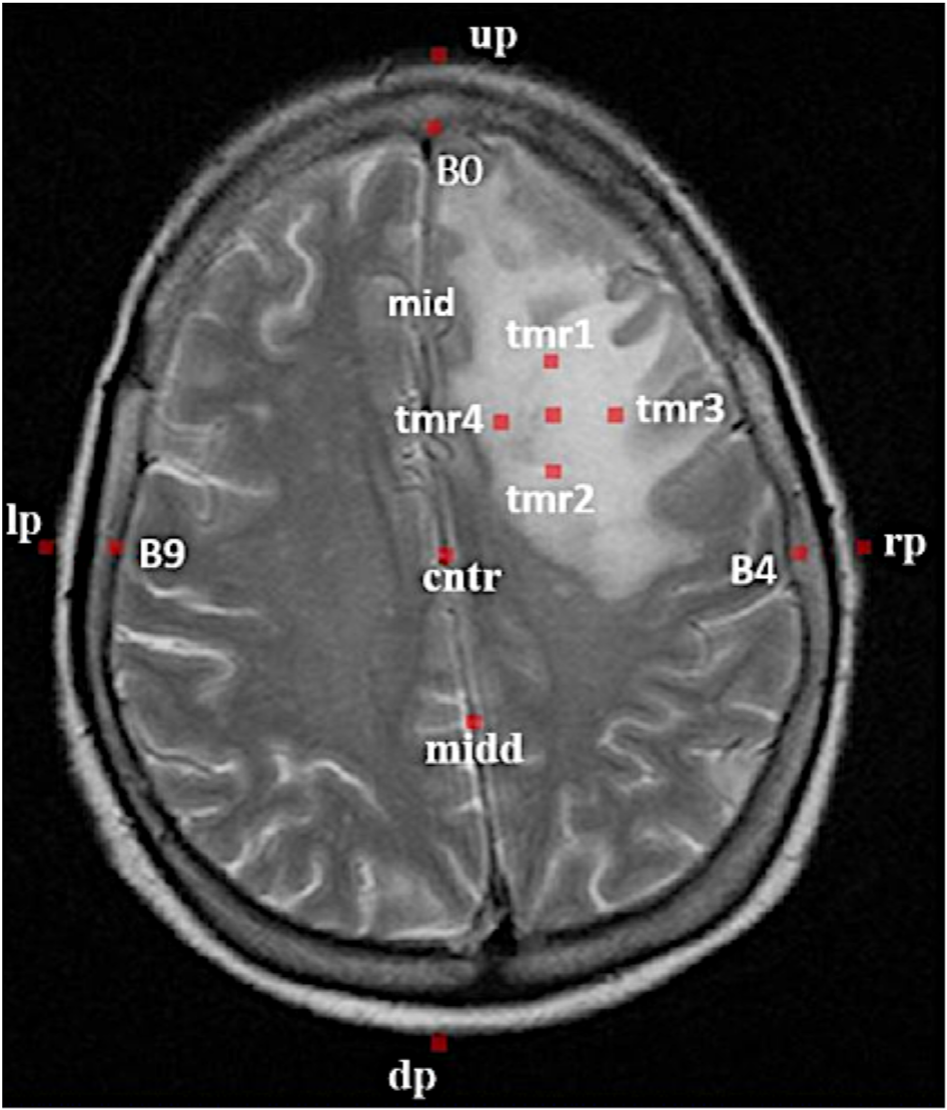
A sample illustration of landmarks on the MR images of a patient.

After that an expert radiologist selected 12 landmarks on each slice, such as vascular and bony landmarks, which also have the most correspondence in every slice of images in different modalities. These landmarks were included up most of the skull (up), downmost of the skull (dp), leftmost of the skull (lp), rightmost of the skull (rp), Falx cerebri (midd), center of the image (cntr), right, left, and up inner parts of the skull (B4), (B9), and (B0). In addition, we considered all four sides around the tumor, including up, down, right, and left inner parts of the tumors (tmr1), (tmr2), (tmr3), and (tmr4), and the tumor center (ctmr), that is, a circle with 1 mm radius in the tumor. (See Figure 5). Moreover, in parallel to this, the radiologist utilized the “Multi‐Atlas Propagation with Enhanced Registration” (MAPER) method, which has been previously validated in various brain image datasets, as a powerful semiautomatic tool for segmenting the whole brain and tumor.^53^

### 4.2 Evaluation metrics

In this study, to assess our registration results, we applied some evaluation metrics including, Dice similarity, target registration error (TRE), Hausdorff, and Jaccard coefficients. Dice coefficient is considered a statistical tool, measuring the similarity of two datasets.^54^ The value of Dice coefficient ranges from 0 to 1, in which 0 and 1 show no overlap and a perfect match, respectively. Here, to compute the Dice coefficient, the segmentation whole brain and tumor were semiautomatically obtained from MAPER,^53^ as a powerful tool that previously has been validated in several brain image datasets^55^ and the results were evaluated by a radiologist. In the registration, TRE is defined based on computing the displacement between the computed and the anatomically selected landmarks, that is, TRE = *X_a_* − *X_b_*. Here, *X_a_* and *X_b_* are the anatomical and computed positions of the landmarks. The displacement was considered as the metric to evaluate the registration algorithms accuracy^56^ that the less TRE, the better registration. Moreover, the Hausdorff metric gauges the distance between two subsets of a metric space from each other, that is, it is the greatest of all the distances from a point in one set to the closest point in the other set.^8^ Finally, the Jaccard similarity coefficient measures diversity as well as the similarity in datasets.^57^

### 4.3 Experimental results

The experimental findings were totally analyzed based on above mentioned four evaluation metrics. There were three baseline approaches, ANT (SyN),^32^, ^47^ NiftReg,^48^, ^49^ and VoxelMorph,^35^ with various strengths. In the current study, the obtained results were compared with these three baselines.

**FIGURE 6:**
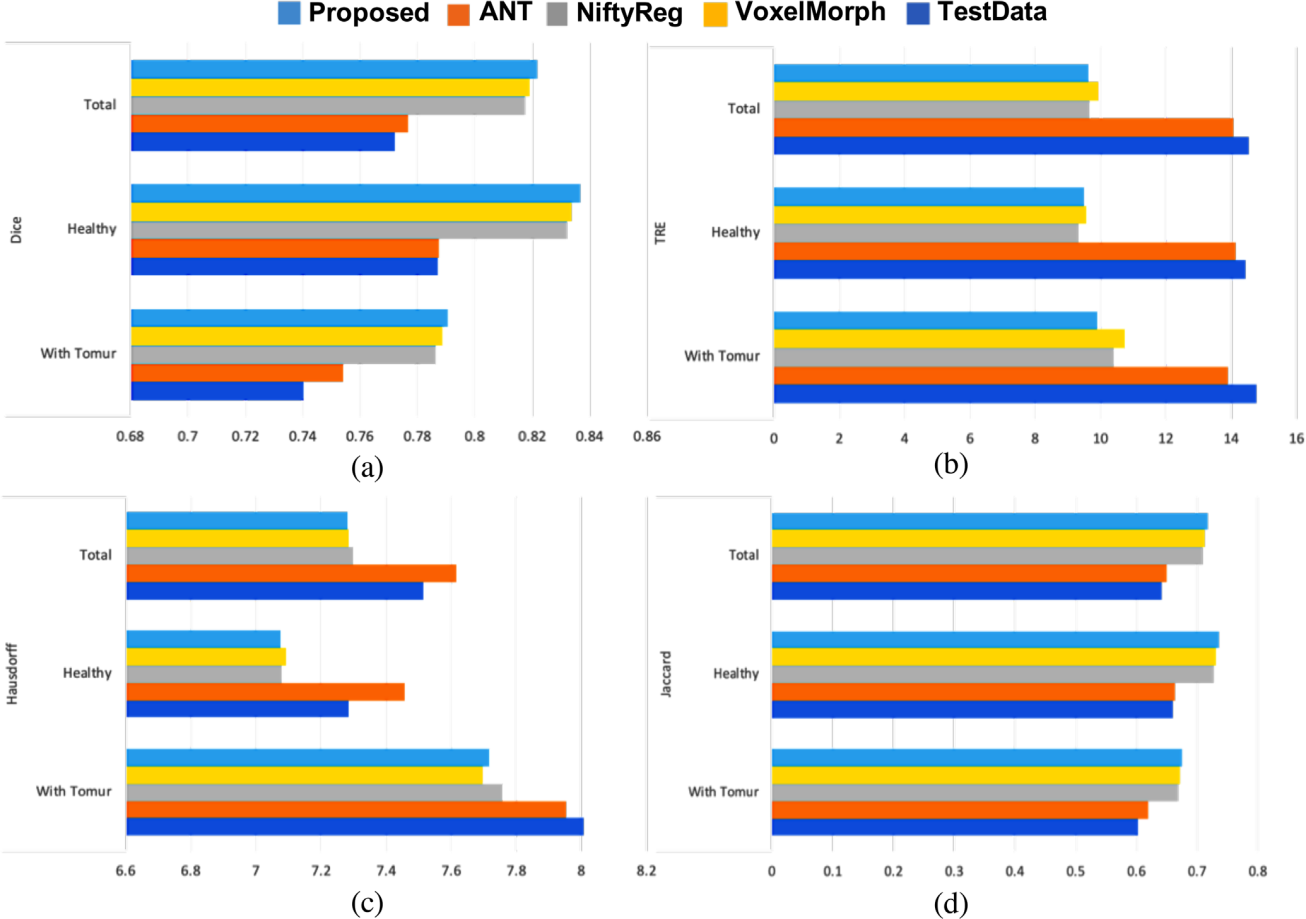
The proposed approach versus the baselines for (a) the Dice; (b) the TRE; (c) the Hausdorff coefficient; (d) the Jaccard coefficient.

Figure 6(a) illustrates the evaluation of the proposed approach versus the other baselines based on the Dice coefficient. Due to the range of dice, that is, [0, 1], the higher dice, the better registration. The proposed model is the most successful than the baselines. Also, Figure 6(b) illustrates the evaluation of the proposed model versus the baselines based on the TRE in millimeter, which is known most approved metric in the registration.^58^, ^59^ Because of the range of the TRE, that is, [0,∞], the lower TRE also results in better registration. The proposed model is more successful in the baselines, and there is also an undeniable enhancement against the row test dataset.

According to Figure 6(c), the proposed model versus the baselines based on the Hausdorff coefficient is better. Because of the range of the Hausdorff coefficient, that is, [0,∞], the lower Hausdorff, results in better registration. Figure 6(d) shows evaluating the proposed model versus the baselines based on the Jaccard coefficient that is a common metric in segmentation and registration. Due to the range of the Jaccard coefficient, that is, [0, 1], the higher Jaccard can result in the better registration that the proposed model is also more successful than the other baselines.

In addition to presenting the results of the models, all results obtained are shown in Table 3, completely. As observed, the proposed approach outperforms the other baselines, as indicated by the largest bin size in MI (64‐bins) and a learning rate of 10^−4^. The time taken by each approach is presented in Table 3. Although the proposed approach requires more time compared to the other baselines, this is acceptable considering that the proposed network is larger and has more parameters. However, it's worth noting that the time of 203 ms is less than a quarter of a second, which is highly favorable for real‐time and clinical applications.

**TABLE 3:** The results of all baselines and the proposed approach with std.

		ANT(SyN)	NiftyReg	VoxelMorph	Proposed
	With tumor	0.71 (0.03)	0.73 (0.06)	0.76 (0.04)	0.80 (0.04)
Dice	Healthy	0.74 (0.10)	0.81 (0.11)	0.82 (0.12)	0.86 (0.06)
	With tumor	13.91 (2.67)	12.38 (2.49)	12.71 (2.55)	9.89 (1.49)
TRE	Healthy	14.13 (3.18)	11.64 (3.31)	11.81 (3.32)	9.50 (1.27)
	With tumor	9.53 (1.74)	9.76 (1.72)	9.70 (1.41)	7.15 (0.80)
Hausdorff	Healthy	7.70 (1.20)	7.84 (1.13)	7.93 (1.50)	7.02 (1.10)
	With tumor	0.62 (0.15)	0.65 (0.18)	0.67 (0.18)	0.75 (0.20)
Jaccard	Healthy	0.66 (0.15)	0.71 (0.12)	0.70 (0.13)	0.77 (0.16)
Time(ms)		299 (± 5 ms)	299 (± 5 ms)	200(± 6 ms)	203(± 5 ms)

**FIGURE 7:**
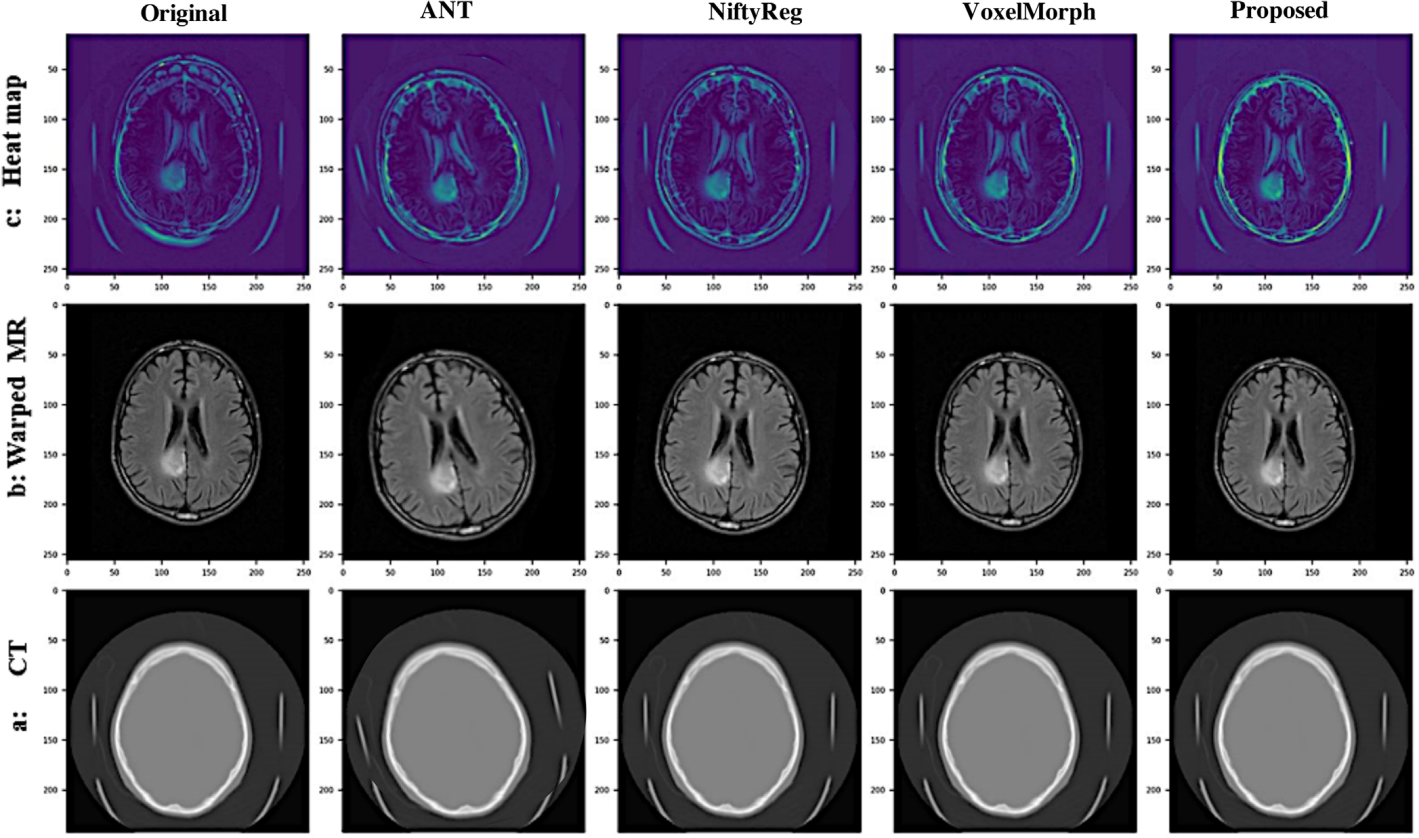
An example of data set images, registered based on the basslines and proposed model, a: CT scans in three baselines and proposed approach, b: MRI warped image obtained by CNN, and c: the heat map, showing the difference between CT and warped MRI.

Figure 7 illustrates an example from the test dataset that has been registered using the baselines and the proposed model. The figure consists of three rows, with each row representing different visualizations. In the first row, a difference heatmap between CT and warped MR images is displayed. The second row shows the warped MR images and the corresponding CT scans. The third row presents the original images and the registered images obtained using ANT, NiftyReg, VoxelMorph, and the proposed model, displayed from left to right in the first to fourth columns, respectively. The fifth column represents the registration image produced by the proposed model. In the heatmap, the sharper points are highlighted in yellow, indicating larger differences. Based on the visual comparison of the images, it can be concluded that the proposed model achieves the best registration performance.

## 5 DISCUSSION

When the entire tumor is not directly visible, deducing its 3D position from image data becomes challenging, as well as through physical examination. Therefore, in treatment planning, radiation oncologists routinely utilize image registration. The literature highlights four main objectives of image registration in treatment planning, which are as follows: (1) Imposing inherent constraints on the definition of tumor volumes in cases such as prostate and brain tumors due to the limitations of CT scanning.^60^ The inferior edge of the prostate, as determined using CT, differed significantly from that determined using urethrogram. These results can be of great utility in the treatment‐planning system for prostate cancer. (2) MRI provides more comprehensive information than CT for accurate detection of the tumor region. Despite the advantages of MRI over CT in visualizing soft tissues, oncologists prefer to utilize image registration techniques to mitigate the inherent magnetic distortion present in MR images, which could otherwise lead to treatment‐planning errors. (3) In cases where cytoreductive chemotherapy is administered, the planning CT scan may not fully visualize the entire tumor. Therefore, there is a need to accurately transfer the tumor from the pre‐chemotherapy scan to the post‐chemotherapy scan, which is a complex process when done manually. (4) Surgical procedures can sometimes result in the tumor not being visible on the planning CT scan. However, for precise radiation therapy targeting the close surgical margin, it is crucial to determine the original tumor location. This can be effectively achieved using image registration techniques.^61^

However, image registration techniques have certain limitations, such as being timeconsuming and heavily reliant on the expertise of the user for accurate results.^6^ Additionally, supervised learning‐based approaches require large datasets, which are challenging to obtain in the field of medical imaging. Moreover, these approaches often rely on ground‐truth datasets, which can be difficult to obtain and may contain errors due to the fatigue of radiologists who are busy with clinical work.^62^ Therefore, unsupervised image registration methods have gained attention as they do not rely heavily on expert annotations of groundtruth data. Many researchers have focused on developing unsupervised methods, which have shown promising results, particularly in unimodal registration tasks. However, in clinical applications, multimodal registration is often necessary to utilize the valuable information from different imaging modalities such as CT and MRI.^6^

Although unsupervised learning addresses the problem of ground‐truth, it has a difficulty for the purpose of evaluation. Some common metrics, for example, the Normalized Cross‐Correlation and Mean Square Distance, which work perfectly for unimodal cross/co‐registration, are not proper for the case of the current study as this study works on multimodal images that have completely different distribution functions. Hence, we applied some more complicated metrics such as MI, which is vectorized and then implemented in the differentiable form to generate full or sub‐gradients, which are necessary for the gradient‐decent back‐propagation learning phase.^35^

The performance of our proposed model shows promising results that are comparable to recent state‐of‐the‐art models. The current study is conducted on the image registration of two modality images, MRI and CT, using multimodal CT/MR affine co‐registration, a deep unsupervised model based on CNN with loss functions MI, leading to delineating the tumor more precisely and accurately due to the use of two different modalities that each one can support some various information, that is, CTs can provide images with high contrast, while MRIs can provide images with high resolutions of the brain.^63^

Here, all CTs and MRIs (both *T*_1_ and *T*_2_ weighted) were randomly taken from various scanners with different manufacture, setups, and also procedures. Therefore, there is heterogeneity in the dataset (resulting in a multimodal and more challenging co‐registration study) that this approach addressed appropriately. In other words, all the limitations in this study change to its novelties, such as multimodal images, co‐registration, different scanners, and different data, as the proposed model could address all of them in proper manner.

Although our pipeline was developed using a local dataset of patients for the registration task as a proof of concept, such methods have numerous potential applications from a clinical practice perspective, especially when applied to different images obtained from the same patient. In the context of radiotherapy, several studies have highlighted significant variations in the targeted volumes of the brain during radiation treatment, which raises the question of treatment replanning to reduce the irradiation of healthy brain tissue in case of tumor reduction or to adapt the treatment for brain tumors that grow during radiation.^64^ With the advent of MR‐guided linacs, daily imaging during radiotherapy treatment becomes possible, and the proposed method could assist in both automatic registration and segmentation for the purpose of replanning. Furthermore, it could enable accurate assessment of the delivered dose in targeted volumes and organs at risk (OARs) by accounting for the observed volume changes. Additionally, as changes in imaging features during treatment are often associated with treatment outcomes in various cancer diseases,^65^ registration of two acquisitions from the same patient at different times allows for an objective and precise evaluation of tumor variations.

In conclusion, we proposed a customized differentiable layer to enhance a deep unsupervised learning‐based model for affine co‐registration of multimodal CT/MR images of the brain. The main goal of this layer was to convert the affine parameters generated by the network into a dense displacement field. By incorporating this affine‐to‐field layer, we were able to introduce regularization in the STN to penalize unrealistic affine transformations produced by the network. This regularization term was crucial and without it, achieving reliable affine registration would not have been possible. Since affine registration operates on a global scale, having an extended receptive field is essential for achieving high performance. However, deepening the network increases the risk of overfitting, especially when dealing with affine transformations that have a small number of degrees of freedom and limited datasets that are typical in medical applications. The utilization of the regularization module proved to be highly beneficial for deepening the network in affine registration tasks, as it effectively prevented overfitting. Consequently, we were able to construct deeper networks with a wider receptive field, leading to significant improvements in the registration performance. Based on the experimental results, the proposed approach, due to more bins in MI (64) and better learning rate (10^−14^), performed better than the other baselines that are also designed based on unsupervised CNN model, that is, the proposed approach obtains the best evaluation metrics, especially TRE and Dice coefficient, which are the most reliable metrics in the realm of image registration than the other baselines, and this model can register a pair of CT/MR based on the affine technique at 203 ms (std: ±5), which is valuable for the real‐time clinical application, with an acceptable TRE.

Meanwhile, it is worth discussing the limitations related to the dataset and evaluation methods, as well as potential future directions that may be of interest to the readers. The primary limitation we encountered in conducting this study was the scarcity of suitable datasets and baseline approaches for evaluating the method. Our study focuses on coregistration, specifically the registration of a patient's own images. This unique multimodal dataset presented a challenge as we had to collect the data ourselves, making it the first of its kind. Furthermore, deep unsupervised co‐registration on multimodal data has not been extensively studied due to the limitations of multimodal similarity metrics like MI. These metrics lack differentiable implementations, which prevents their direct use in deep networks that require gradient‐based optimization. However, we addressed this issue in the base network. Nevertheless, there is still a need for further exploration and investigation of alternative terms in conjunction with the affine‐to‐field layer to assess their impact on affine registration. In summary, moving forward, future research could focus on exploring different regularizer terms in conjunction with the affine‐to‐field layer to examine their efficacy in improving affine registration performance.

## AUTHOR CONTRIBUTIONS

Samaneh Abbasi, Alireza Mehdizadeh, and Meysam Tavakoli developed the idea; Samaneh Abbasi wrote the first edition of the manuscript; Meysam Tavakoli, wrote the revised manuscript; Samaneh Abbasi, Hamid Reza Boveiri, and Meysam Tavakoli developed computational tools, and wrote the manuscript; Mohammad Amin Mosleh Shirazi contributed the data; Meysam Tavakoli, Hamid Reza Boveiri, Raouf Khayami, Reza Javidan, and Alireza Mehdizadeh conceived research; Meysam Tavakoli, and Alireza Mehdizadeh oversaw all aspects of the projects.

## CONFLICT OF INTEREST STATEMENT

The authors have no relevant conflict of interest to disclose.
